# Novel Subgroups and Chronic Complications of Diabetes in Middle-Aged and Elderly Chinese:A Prospective Cohort Study

**DOI:** 10.3389/fendo.2021.802114

**Published:** 2022-01-26

**Authors:** Fei Wang, Ruizhi Zheng, Ling Li, Min Xu, Jieli Lu, Zhiyun Zhao, Mian Li, Tiange Wang, Shuangyuan Wang, Yufang Bi, Yu Xu, Guang Ning, Weimin Cai

**Affiliations:** ^1^ Department of Clinical Pharmacy and Pharmaceutical Management, School of Pharmacy, Fudan University, Shanghai, China; ^2^ Department of Endocrine and Metabolic Diseases, Shanghai Institute of Endocrine and Metabolic Diseases, Ruijin Hospital, Shanghai Jiao Tong University School of Medicine, Shanghai, China; ^3^ Shanghai National Clinical Research Center for Metabolic Diseases, Key Laboratory for Endocrine and Metabolic Diseases of the National Health Commission of the PR China, Shanghai Key Laboratory for Endocrine Tumor, State Key Laboratory of Medical Genomics, Ruijin Hospital, Shanghai Jiao Tong University School of Medicine, Shanghai, China; ^4^ Department of Pharmacy, Ruijin Hospital, Shanghai Jiao Tong University School of Medicine, Shanghai, China

**Keywords:** type 2 diabetes, cluster analysis, diabetic complications, metabolic associated fatty liver disease, subclinical atherosclerosis

## Abstract

**Background:**

Diabetes mellitus, especially type 2 diabetes mellitus (T2DM), is regarded as highly heterogeneous. Novel diabetes phenotypes by cluster analysis have been proposed in Europeans but may show different cluster features in Asians. The applicability of cluster analysis in middle-aged and elderly Chinese community T2DM patients needs further investigation.

**Methods:**

Participants were recruited from Jiading community in Shanghai, China. We adopted k-means cluster analysis in 1130 patients (aged ≥ 40 years) with newly-diagnosed T2DM at baseline. Cluster analysis was performed based on seven variables, including fasting plasma glucose, 2 hours postprandial blood glucose, age at diagnosis, body mass index, hemoglobin A1c, homoeostatic model assessment estimates of β-cell function and insulin resistance. All subjects were re-examined at 4.4 years later. Metabolic associated fatty liver disease was diagnosed using B-ultrasound, hepatic fibrosis by non-invasive scores, renal and cardiovascular status by subclinical biomarkers. Multivariable logistic regression models were used to compare the risks of complications between clusters.

**Results:**

Patients were classified into 4 clusters. 381 (33.7%), 456 (40.4%), 87 (7.7%), and 206 (18.2%) patients were separately assigned to mild age-related diabetes (MARD), mild obesity-related diabetes (MOD), severe insulin-deficient and insulin-resistant diabetes (SIDRD), or severe obesity-related and insulin-resistant diabetes (SOIRD), respectively. Participants in MARD, SOIRD, and SIDRD clusters were associated with significantly increased risks of different complications. SOIRD and SIDRD showed novel features in Chinese T2DM patients that were different from those in Europeans.

**Conclusions:**

The refined diabetes phenotypic approach was applicable to Chinese middle-aged and elderly T2DM patients. Patients in different clusters presented significantly different characteristics, progression of metabolic features, and risks of diabetic complications.

## Introduction

The number of adults with diabetes has progressively increased from 108 million in 1980 to 536.6 million in 2021 worldwide (1, 2). With a dramatic change in lifestyle in recent 30 years, China becomes the country with the largest number of patients affected by diabetes. A national survey in 2015-2017 showed that the prevalence of diabetes among Chinese people aged ≥ 18 years was 12.8%, but the rate of diabetes control was low (3). Poor control of blood glucose is associated with higher risk of incident diabetic complications, leading to a huge burden on the patients.

Nowadays, the most widely used diagnosis of diabetes is based on fasting and post-load glucose as well as hemoglobin A1c (HbA1c) levels defined by the American Diabetes Association (ADA) criteria (4). However, such criteria could not provide precision treatment recommendations for diabetes, which has heterogeneous physiopathology (4, 5). Recently, a novel approach has been proposed to classify diabetes into 5 subgroups with different characteristics (6): two mild subgroups with good metabolic control and few diabetes-related complications, and three severe subgroups with poor glucose control and increased risks of clinical outcomes. Replications of novel diabetes subgroups have been observed in cohorts from north Europe, USA, and Asia (7–9). The Chinese population is more likely to have a fragile β-cell function and more susceptible to the effects of obesity on metabolic factors (10, 11). Chinese patients with type 2 diabetes mellitus (T2DM) present with different characteristics from those in other populations (12). Additionally, 2h post-load plasma glucose (2hPG) is an important predictive factor of clinical outcomes in Chinese adults independent of fasting plasma glucose (FPG) and HbA1c (13), but 2hPG was not considered in any cluster analysis of diabetes in aforementioned studies (6, 14, 15).

Furthermore, most reports were cross-sectional (7, 14) and few studies have examined such classification method among Chinese T2DM patients. In the current study, we aimed to examine the novel subgroups of diabetes in a cohort of middle-aged and elderly Chinese using variables reported in previous studies plus FPG and 2hPG at baseline (6), to evaluate the changes of specific metabolic markers during follow-up, and to assess the risks of developing diabetes-related complications among different clusters.

## Methods

### Study Design and Participants

In the present study, participants with newly-diagnosed T2DM were included from a prospective cohort study of 10,375 adults aged ≥ 40 years in Jiading District, the suburb of Shanghai, China. The details of the study design have been reported previously (16, 17). Briefly, the baseline examination was conducted between March and August in 2010. Participants were interviewed face to face by well-trained staff with a comprehensive evaluation including a standard questionnaire, anthropometric measurements, blood and urine sampling, and biochemical determination according to a standard protocol. During August 2014 and May 2015, participants were invited for a follow-up examination to reassess their health conditions.

The study protocol was approved by the Institutional Review Board of Ruijin Hospital, Shanghai Jiaotong University School of Medicine. Informed consent was provided by each participant in advance.

### Data Collection

A standard questionnaire including demographic characteristics, history of chronic diseases, medications, and lifestyle factors was administrated by a face-to-face interview. Current smoking and drinking were defined as smoking cigarettes or consuming alcohol regularly during past 6 months. Physical activity was inquired and evaluated using metabolic equivalent hours per week (MET-h/wk) by the short form of the International Physical Activity Questionnaire (IPAQ) (18). Being physically active was defined as at least 7.5 MET-h/wk (19). Anthropometric measurements, including body height, weight, and waist and hip circumferences were measured according to a standard protocol. Body mass index (BMI) was calculated as body weight divided by height squared (kg/m^2^). Blood pressure (BP) was measured three times after at least 5-minute sitting rest using a calibrated automatic electronic device (OMRON Model HEM-752), with alcohol, tea, coffee, and exercise being strictly avoided 30 minutes before measurement. The average of 3 measurements was used for analysis. Blood samples were collected in early morning with an overnight fast for at least 10 hours. All participants without a diabetes history underwent a standard 75-g oral glucose tolerance test (OGTT), and blood samples were collected at 0 h and 2 h. Plasma glucose was measured using the glucose oxidase method on an autoanalyzer (Modular P800; Roche, Basel, Switzerland). HbA1c was determined by high-performance liquid chromatography using the VARIANT II Hemoglobin Testing System (Bio-Rad Laboratories). Biochemical parameters including total cholesterol (TC), triglycerides (TG), low-density lipoprotein cholesterol (LDL-c), high-density lipoprotein cholesterol (HDL-c), alanine aminotransferase (ALT), aspartate aminotransferase (AST), γ-glutamyl transferase (GGT), apolipoprotein B (ApoB), apolipoprotein A1 (ApoA1) and albumin were measured with auto analyzers (Modular Analytics P800 and Modular E170; Roche, Basel, Switzerland). Serum creatinine (SCr) was measured using the picric acid method (clinical chemistry diagnostic system C16000, Abbott Laboratories, Otawarashi, Japan). A first void spot urine sample was collected in early morning to measure urinary albumin using immunoturbidimetric method (Beijing Atom High-Tech, Beijing, China) and urinary creatinine using Jaffe’s kinetic method (Hitachi 7600-020, Tokyo, Japan).

Ankle-brachial index (ABI), brachial to ankle pulse wave velocity (ba-PWV), and carotid intima-media thickness (CIMT) were measured to evaluate subclinical atherosclerosis. The values of ABI and ba-PWV were obtained by Colin VP-1000 (Model BP203RPE II, form ABI/PWV). The ABI was calculated by the ratio of the dorsal foot or posterior tibial artery systolic BP to the brachial artery systolic BP. The ba-PWV was measured as pulse waves distances (obtained from the brachial and tibial arteries) divided by the transmission time. The CIMT was measured by high-resolution B-mode tomographic ultrasound system (Esaote Biomedica SpA, Italy) with a linear 7.5-MHz transducer. The operator measured CIMT on the far wall of the common carotid arteries at 1.5 cm proximal to the bifurcation. The distance from the leading edge of the first echogenic line to the second at the end of diastole was taken for CIMT. The higher bilateral ba-PWV and CIMT value was used for analysis.

### Definitions of Diabetes and Other Complications

Newly-diagnosed diabetes was defined by FPG ≥ 7.0 mmol/L (126 mg/dL) and/or 2hPG ≥ 11.1 mmol/L (200 mg/dL) during OGTT and/or HbA1c ≥ 6.5% excluding self-reported diabetes or use of diabetes medications (4). Hypertension was defined by BP ≥ 140/90 mmHg (20) and/or using antihypertensive medications within 2 weeks. Dyslipidemia was defined by TC ≥ 6.2 mmol/L (240 mg/dL) and/or LDL cholesterol ≥ 4.1 mmol/L (160 mg/dL) and/or TG ≥ 2.3 mmol/L (200 mg/dL) and/or HDL cholesterol ≤ 1.0 mmol/L (40 mg/dL) and/or taking lipid-lowering drugs (21). Obesity was defined by BMI ≥ 25 kg/m^2^. We estimated β-cell function by the homoeostatic model assessment estimates of β-cell function (HOMA-β) and insulin resistance by the homoeostatic model assessment estimates of insulin resistance (HOMA-IR) (22). Estimated glomerular filtration rate (eGFR) was calculated with serum creatinine levels using the Chronic Kidney Disease Epidemiology Collaboration (CKD-EPI) equation (23). Urinary albumin-creatinine ratio (ACR) was calculated by dividing urinary albumin concentrations (mg/L) by urinary creatinine concentrations (g/L). Chronic kidney disease (CKD) was defined by eGFR < 60 ml/min per 1.73 m^2^ or ACR ≥ 30 mg/g. Subclinical peripheral artery disease was defined as an ABI ≤ 1.0 or >1.3 (24, 25). Elevated CIMT (≥ 0.7 mm) or ba-PWV (≥ 1991 cm/s) was defined as levels within the upper quartile of the study population at baseline.

Metabolic associated fatty liver disease (MAFLD) was defined by diabetes complicated with hepatic steatosis (26). Abdominal ultrasonography was performed to identify fatty liver in all the participants as recommended ^26^ Two trained sonographers who were blinded to both clinical and laboratory data, operated the high-resolution B-mode tomographic ultrasound system (Esaote Biomedica SpA) with a 3.5-MHz probe. According to the international expert consensus statement in 2020 and Chinese Association for the Study of Liver Disease, fatty liver was diagnosed by the presence of at least two of the following findings: (1) diffusely increased echogenicity of the liver relative to the kidney, (2) ultrasound beam attenuation, or (3) poor visualization of intrahepatic structures (27). A third trained sonographer who was also blinded to this study would be asked if the diagnosis of fatty liver was contradicted between the previous two sonographers.

Noninvasive liver fibrosis scores including nonalcoholic fatty liver disease fibrosis score (NFS), Aspartate aminotransferase/platelet ratio index (APRI) and Fibrosis-4 Index (FIB-4) were calculated with routine laboratory variables according to the equations listed below (28–30). The higher probability of fibrosis was defined as NFS ≥ −1.455, FIB-4 ≥ 1.3, or APRI ≥ 0.5.

### Equations


HOMA−β=20×fasting insulin (μIU/mL)/[fasting glucose (mmol/L)−3.5]

HOMA−IR=fasting insulin (μIU/mL)×fasting glucose (mmol/L)/22.5

$$\begin{array}{l} \text{NFS}=-1.675+0.037\times \text{age }(\text{years})+0.094\times {\text{BMI (kg/m}}^{2})+1.13\\ \times \text{impaired fasting glucose or diabetes }(\text{yes}=1,\text{no}=0)+0.99\\ \times \text{AST (U/L)/ALT (U/L)}-0.013\times \text{platelet count (}\times 10^{9}\text{/L)}-0.66\times \text{albumin (g/dL)} \end{array}$$

$$\text{APRI}=[(\text{AST/normal upper limit AST)/platelet count(}\times 10^{9}\text{/L)]}\times 100$$

$$\text{FIB}-4=\text{age (years)}\times \text{AST (U/L)/[platelet count (}\times 10^{9}\text{/L)}\times {\text{ALT(U/L)}}^{1/2}]$$

### Statistical Analysis

In our study, K-means cluster analysis was performed with 7 variables including BMI, age at diagnosis of diabetes, FPG, 2hPG, HbA1c, HOMA-β, and HOMA-IR at baseline. The clustered variables are centered to means of 0 and standard deviations (SDs) of 1. We replicated the clustering approach used by Ahlqvist et al. (6). The K-means cluster analysis was processed with a k value of 4 using the kmeansruns function (runs = 150) in the fpc package in R version 4.0.3. We named the clusters based on the distinct cluster characteristics.

The continuous variables were presented in mean (standard deviation) or median (25% quartile, 75% quartile), and categorical variables were in numbers (%). Comparisons of means and percentages were performed by the ANOVA and Chi-square tests between clusters, respectively. Bonferroni correction was applied to account for multiple comparisons. Skewed variables were log-transformed before analysis. Logistic regression analyses were done to compare the risks of incident diabetes-related complications between clusters, and the cluster with the lowest incidence of complications was used as the reference. The multivariable model was adjusted for sex, lifestyle (smoking status, drinking status, physical activity), family history of diabetes, education status, hypertension and dyslipidemia. Statistical analysis was performed with R version 4.0.3. We judged *P* values less than 0.05 as statistical significance (2-sided).

## Results

After excluding missing data on cluster variables (n = 6), a total of 1130 newly-diagnosed T2DM participants were included for baseline cluster analysis. 688 in 1130 participants with complete cluster variable data underwent the follow-up interview. Participants with baseline self-reported disease history, missing data of biochemical or atherosclerotic cardiovascular measurements, or diabetes complications were further excluded for the association analysis (**Supplementary Figure 1**).

### Cluster Distribution and Characteristics at Baseline

**Figure 1:**
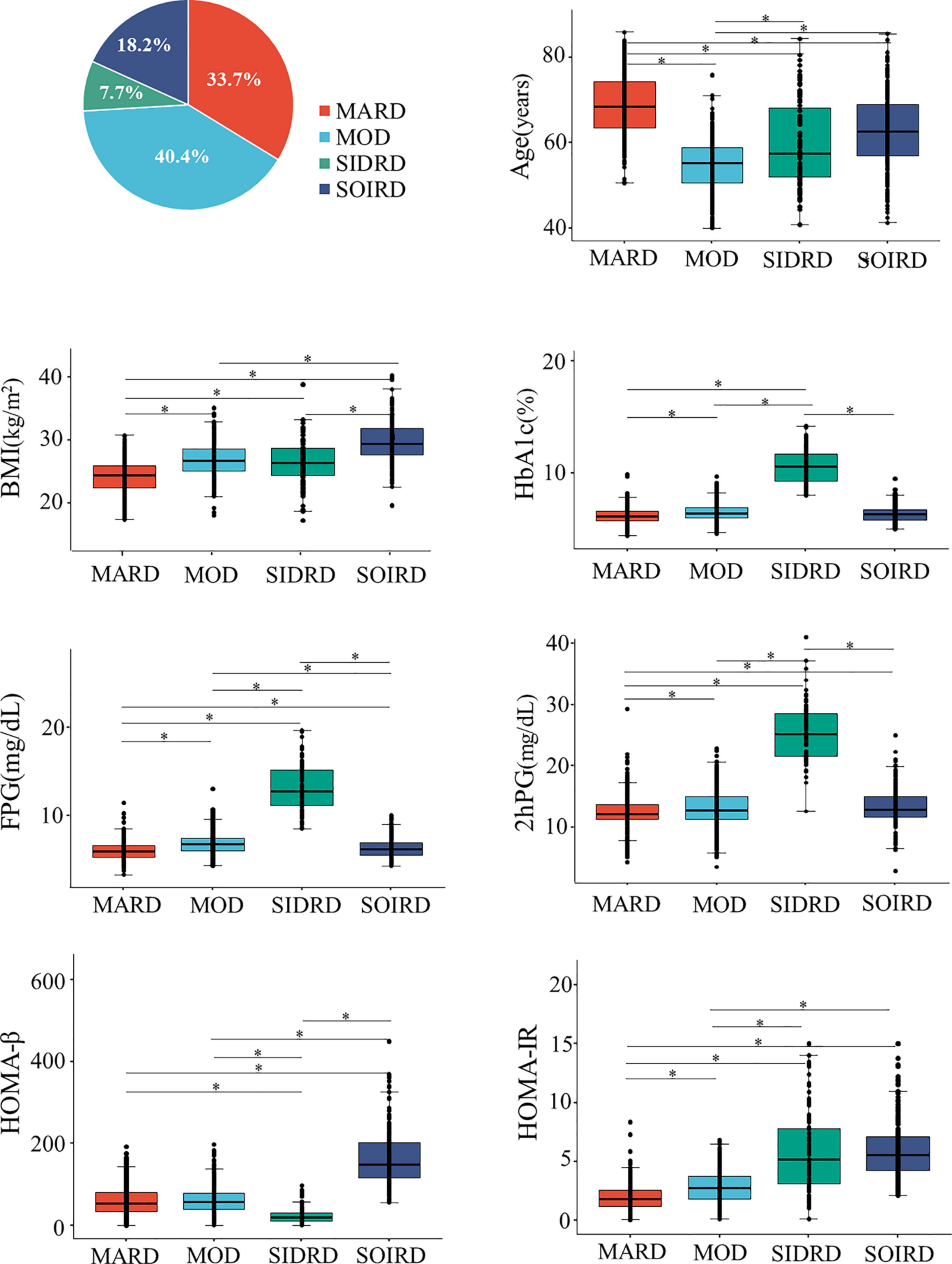
Distributions and cluster characteristics at baseline. *The differences between clusters were compared by using t tests, and the Bonferroni correction was used to adjust the statistical significance level (*P* = 0.05/6). MARD, mild age-related diabetes; MOD, mild obesity-related diabetes; SIDRD, severe insulin-deficient and insulin-resistant diabetes; SOIRD, severe obesity-related and insulin-resistant diabetes; BMI, body mass index; HbA1c, hemoglobin A1c; FPG, fasting plasma glucose; 2hPG, 2 hours postprandial blood glucose; HOMA-β, homoeostatic model assessment estimates of β-cell function; HOMA-IR, homoeostatic model assessment estimates of insulin resistance.

Participants were classified into 4 diabetes subgroups based on 7 variables measured at baseline (**Figure 1** and **Supplementary Material**). They were categorized as mild age-related diabetes (MARD), mild obesity-related diabetes (MOD), severe insulin-deficient and insulin-resistant diabetes (SIDRD) and severe obesity-related and insulin-resistant diabetes (SOIRD) respectively.

381 (33.7%) patients were assigned to the MARD cluster. Participants assigned to MARD had the oldest age at diagnosis (mean 68.8 years old), lowest BMI (mean 24.2 kg/m^2^), and modest metabolic disturbances (mean HbA1c 6.2%, median HOMA-β 52.9, HOMA-IR 1.8). The MOD cluster makes up the largest proportion among diabetes patients (n = 456, 40.4%). Individuals in the MOD cluster were characterized by younger age at diagnosis (mean 54.8 years old) and obesity (mean BMI 26.9 kg/m^2^), but with moderate insulin release and insulin resistance status (median HOMA-β 57.0, HOMA-IR 2.7). Patients in the SIDRD cluster (n = 87, 7.7%) manifested serious insulin deficiency and insulin resistance (median HOMA-β 19.3, HOMA-IR 5.2), and the worst glucose control (mean HbA1c 10.6%, FPG 13.0 mmol/L, 2hPG 25.4 mmol/L). 206 (18.2%) individuals were assigned to the SOIRD cluster who were characterized as having the highest BMI (mean 29.8 kg/m^2^), the most serious insulin resistance, and the highest insulin release level (median HOMA-β 148.9, HOMA-IR 5.6).

Anthropometric and clinical data of the participants at baseline are presented in **Table 1** by clusters. Participants in the MARD cluster presented with the lowest proportion of family history of diabetes (8.7%), lower level of LDL cholesterol (3.27 ± 0.88 mmol/L), TC (5.51 ± 1.01 mmol/L) and TG (median 1.53 mmol/L), and the highest level of HDL cholesterol (1.37 ± 0.36 mmol/L) than those in other clusters. Meanwhile, participants in the SIDRD cluster had higher proportion of smoking (41.3%), drinking (35.6%) than those of the other clusters and SIDRD was the only cluster with a larger proportion of male (60.9%) than the other clusters.

**Table 1:** Patients’ characteristics at baseline among the 4 clusters.

Characteristics	Total	MARD	MOD	SIDRD	SOIRD	*P* value
No. of participants, n (%)	1130 (100.0)	381 (33.7)	456 (40.4)	87 (7.7)	206 (18.2)	<0.001
Age (yr)	61.4 (9.6)	68.8 (7.2)	54.8 (6.1)	59.9 (10.2)	62.9 (9.0)	<0.001
Male, n (%)	466 (41.2)	158 (41.5)	196 (43)	53 (60.9)	59 (28.6)	<0.001
BMI (kg/m^2^)	26.5 (3.5)	24.2 (2.7)	26.9 (2.7)	26.4 (3.6)	29.8 (3.5)	<0.001
FPG (mmol/L)	7.0 (2.2)	6.1 (1.0)	6.9 (1.2)	13.0 (2.7)	6.4 (1.1)	<0.001
2hPG (mmol/L)	13.8 (4.7)	12.3 (3.2)	13.0 (3.2)	25.4 (4.7)	13.4 (3.2)	<0.001
HbA1c (%)	6.7 (1.4)	6.2 (0.7)	6.5 (0.8)	10.6 (1.6)	6.4 (0.7)	<0.001
HOMA-IR	2.8 (1.7, 4.2)	1.8 (1.2, 2.6)	2.7 (1.8, 3.8)	5.2 (3.1, 7.8)	5.6 (4.2, 7.1)	<0.001
HOMA-β	61.4 (36.6, 102.7)	52.9 (34.8, 80.8)	57.0 (38.7, 78.9)	19.3 (11.3, 29.8)	148.9 (116.8, 202.7)	<0.001
Smoking, n (%)	249 (22.0)	58 (15.2)	130 (8.5)	36 (41.3)	25 (12.1)	<0.001
Drinking, n (%)	229 (20.3)	61 (16.5)	113 (24.8)	31 (35.6)	24 (11.7)	<0.001
Physical activity (METs-h/wk)	23.1 (0.0, 53.4)	23.10 (4.7, 42.0)	28.0 (0.0, 132.0)	23.1 (3.6, 92.8)	23.1 (0.0, 46.2)	<0.001
Family history of diabetes, n (%)	160 (14.2)	33 (8.7)	86 (18.9)	15 (17.2)	26 (12.6)	0.001
Systolic blood pressure (mmHg)	149.0 (19.4)	149.3 (18.1)	146.8 (19.7)	149.3 (19.1)	151.5 (20.9)	0.027
Diastolic blood pressure (mmHg)	85.0 (10.7)	81.6 (10.0)	87.6 (10.5)	86.9 (10.6)	84.9 (11.0)	<0.001
LDL cholesterol (mmol/L)	3.33 (0.91)	3.27 (0.88)	3.33 (0.90)	3.39 (1.10)	3.41 (0.90)	0.345
HDL cholesterol (mmol/L)	1.27 (0.31)	1.37 (0.36)	1.23 (0.28)	1.20 (0.29)	1.22 (0.27)	<0.001
Total cholesterol (mmol/L)	5.59 (1.10)	5.51 (1.01)	5.57 (1.11)	5.96 (1.51)	5.60 (1.02)	0.007
Triglycerides (mmol/L)	1.75 (1.26, 2.27)	1.53 (1.11, 2.07)	1.79 (1.24, 2.46)	2.23 (1.48, 3.37)	2.00 (1.54, 2.58)	<0.001

Data are expressed as mean (SD) for variables with normal distribution, median (IQR) for variables with skewed distribution, and n (%) for categorical variables.

BMI, body mass index; FPG, fasting plasma glucose; 2hPG, 2 hours post-load plasma glucose; HbA1c, hemoglobin A1c; HOMA-β, homoeostatic model assessment estimates of β-cell function; HOMA-IR, homoeostatic model assessment estimates of insulin resistance; LDL, low-density lipoprotein; HDL, high-density lipoprotein; MARD, mild age-related diabetes; MOD, mild obesity-related diabetes; SIDRD, severe insulin-deficient and insulin-resistant diabetes; SOIRD, severe obesity-related and insulin-resistant diabetes.

### Risk of Developing Diabetes-Related Clinical/Subclinical Complicationsby Clusters

**Table 2** shows the incidences and risks of developing diabetes-related complications at follow-up by clusters. During a median of 4.4 years, the risk of developing elevated ba-PWV was higher in cluster MARD (OR 2.60; 95% CI 1.15-6.35) compared with SOIRD. The risk of developing abnormal ABI was higher in cluster SOIRD (OR 3.46; 95% CI 1.54-8.04) compared with MARD. Patients in MARD (OR 3.40; 95% CI 1.64-7.41), SIDRD (OR 3.93; 95% CI 1.40-10.58) and SOIRD (OR 2.70; 95% CI 1.13-6.52) clusters all showed higher risks of CKD compared with MOD.

**Table 2:** Risks of diabetes-related complications in 4 clusters at follow-up.

	N	n (%)	OR (95% CI)	
			Model 1	Model 2
ba-PWV ≥ 1991 (cm/s)				
MARD	126	28 (22.2)	2.26 (1.01-5.44)	2.60 (1.15-6.35)*
MOD	241	28 (11.6)	1.10 (0.50-2.65)	1.21 (0.54-2.93)
SIDRD	37	8 (216)	2.45 (0.82-7.28)	2.85 (0.94-8.59)
SOIRD	84	9 (10.7)	Reference	Reference
ABI ≤ 1.0 or > 1.3				
MARD	162	13 (8.0)	Reference	Reference
MOD	229	19 (8.3)	0.98 (0.44-2.25)	1.00 (0.45-2.30)
SIDRD	47	4 (8.5)	1.05 (0.28-3.29)	1.08 (0.28-3.41)
SOIRD	91	19 (20.9)	3.41 (1.55-7.79)^**^	3.46 (1.54-8.04)^**^
CIMT ≥ 0.7 (mm)				
MARD	137	93 (67.9)	0.99 (0.60-1.63)	0.99 (0.60-1.63)
MOD	239	160 (66.9)	Reference	Reference
SIDRD	45	31 (68.9)	0.98 (0.49-2.05)	0.97 (0.48-2.04)
SOIRD	84	61 (72.6)	1.24 (0.70-2.25)	1.25 (0.70-2.28)
ACR ≥ 30 (mg/g) or eGFR < 60 (ml/min/1.73m^2^)				
MARD	160	31 (19.4)	3.37 (1.63-7.33)^**^	3.40 (1.64-7.41)^**^
MOD	217	15 (6.9)	Reference	Reference
SIDRD	44	10 (22.7)	4.00 (1.44-10.72)^**^	3.93 (1.40-10.58)^**^
SOIRD	86	14 (16.3)	2.85 (1.19-6.86)^*^	2.70 (1.13-6.52)^*^
MAFLD				
MARD	123	15 (12.2)	Reference	Reference
MOD	121	37 (30.6)	4.45 (2.07-10.10)^***^	4.74 (2.19-10.86)^***^
SIDRD	20	3 (15.0)	1.09 (0.16-4.63)	1.10 (0.16-4.75)
SOIRD	16	7 (43.8)	5.20 (1.48-17.82)^**^	5.06 (1.44-17.35)^**^
FIB-4 ≥ 1.3				
MARD	60	48 (80.0)	4.11 (1.36-12.95)^*^	4.10 (1.32-13.31)^*^
MOD	156	87 (55.8)	1.26 (0.51-3.13)	1.23 (0.48-3.12)
SIDRD	25	13 (52.0)	Reference	Reference
SOIRD	39	25 (64.1)	1.76 (0.57-5.55)	1.90 (0.60-6.14)
APRI ≥ 0.5				
MARD	183	30 (16.4)	1.14 (0.63-2.04)	1.11 (0.61-1.99)
MOD	253	38 (15.0)	Reference	Reference
SIDRD	46	7 (15.2)	1.12 (0.42-2.64)	1.20 (0.45-2.86)
SOIRD	79	16 (20.3)	1.43 (0.67-2.92)	1.50 (0.70-3.09)
NFS ≥ -1.455				
MARD	35	26 (74.3)	1.06 (0.41-2.84)	0.93 (0.35-2.55)
MOD	91	60 (65.9)	Reference	Reference
SIDRD	18	14 (77.8)	1.66 (0.51-6.47)	2.11 (0.63-8.56)
SOIRD	23	16 (69.6)	1.14 (0.39-3.72)	1.31 (0.44-4.30)

***p < 0.001, **p < 0.01, *p < 0.05.

N, the number of individuals in the cluster at follow-up; n, the number of individuals with clinical/subclinical outcomes.

Model 1 was adjusted for gender, lifestyle (including smoking status, drinking status, physical activity), family history of diabetes, and education status. Model 2 was further adjusted for hypertension and dyslipidaemia.

OR, odds ratio; CI, confidence interval; MAFLD, metabolic associated fatty liver disease; FIB-4, fibrosis 4 score; ACR, albumin-to-creatinine ratio; eGFR, estimated glomerular filtration rate; ba-PWV, brachial-ankle pulse wave conduction velocity; ABI, ankle brachial index; CIMT, carotid intima - media thickness; MARD, mild age-related diabetes; MOD, mild obesity-related diabetes; SIDRD, severe insulin-deficient and insulin-resistant diabetes; SOIRD, severe obesity and insulin-resistant diabetes.

A total of 62 (22.1%) patients developed MAFLD. The incidence of newly-diagnosed MAFLD at follow-up was 12.2%, 30.6%, 15.0%, and 43.8% in MARD, MOD, SIDRD, and SOIRD, respectively. Participants assigned to the MOD (OR 4.74; 95% CI 2.19-10.86) and the SOIRD cluster (OR 5.06; 95% CI 1.44-17.35) were significantly associated with higher risk of incident MAFLD compared with the MARD cluster. Compared with SIDRD, MARD was associated with an increased risk of liver fibrosis defined by FIB-4 ≥ 1.3 (OR 4.10; 95% CI 1.32-13.31).

### Changes in Clinical Metabolic Biomarker

**Figure 2:**
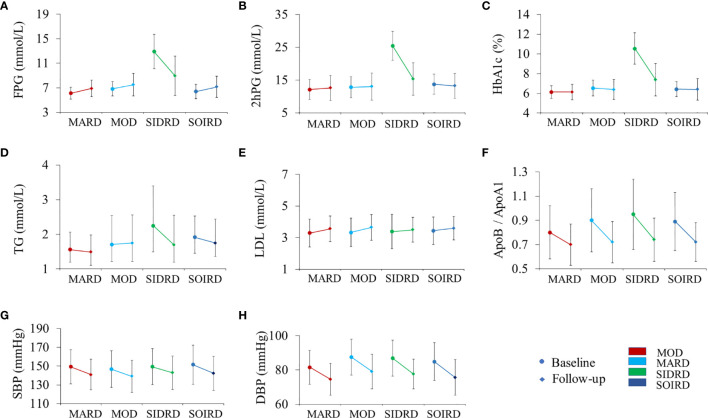
Changes in the metabolic biomarkers during follow-up by clusters. Plots show levels of FPG **(A)**, 2hPG **(B)**, HbA1c **(C)**, TG **(D)**, LDL **(E)**, ApoB/ApoA1 **(F)**, SBP **(G)**, and DBP **(H)**. Levels are shown for patients with data available at both baseline (circles) and follow-up (diamond). Data are expressed as mean (SD) for variables with normal distribution or median (IQR) for variables with skewed distribution. *P* values for paired t-test between baseline and follow-up by clusters are presented in **Supplementary Table 2**. MARD, mild age-related diabetes; MOD, mild obesity-related diabetes; SIDRD, severe insulin-deficient and insulin-resistant diabetes; SOIRD, severe obesity-related and insulin-resistant diabetes; FPG, fasting plasma glucose; 2hPG, 2 hours postprandial blood glucose; HbA1c, hemoglobin A1c; TG, triglyceride; LDL, low density lipoprotein; ApoB, apolipoprotein B; ApoA1, apolipoprotein A1; SBP, systolic blood pressure; DBP, diastolic blood pressure.

**Figure 2** shows the changes of blood glucose, serum lipids, and blood pressure from baseline to follow-up by clusters. The levels of glycemic parameters (FPG, 2hPG and HbA1c) were significantly decreased in SIDRD. The levels of FPG were significantly increased in the clusters of MARD, MOD and SOIRD, while 2hPG showed no significant change in these clusters after 4.4 years. TC and ApoB/ApoA1 ratio were also decreased dramatically during the follow-up period (**Supplementary Table 2**). The SBP and DBP levels declined in all the clusters.

**Figure 3:**
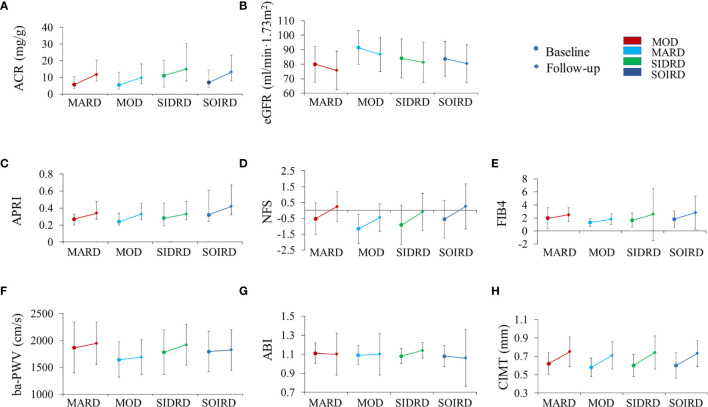
The progression of chronic complications during follow-up by clusters. Plots show levels of ACR **(A)**, eGFR **(B)**, APRI **(C)**, NFS **(D)**, FIB4 **(E)**, ba-PWV **(F)**, ABI **(G)**, and CIMT **(H)**. Levels are shown for patients with data available at both baseline (circles) and follow-up (diamond). Data are expressed as mean (SD) for variables with normal distribution or median (IQR) for variables with skewed distribution. *P* values for paired t-test between baseline and follow-up by clusters are presented in **Supplementary Table 3**. MARD, mild age-related diabetes; MOD, mild obesity-related diabetes; SIDRD, severe insulin-deficient and insulin-resistant diabetes; SOIRD, severe obesity-related and insulin-resistant diabetes. ACR, urinary albumin-creatinine ratio; eGFR, Estimated glomerular filtration rate; APRI, aspartate amino transferase/platelet ratio index; NFS, fatty liver disease fibrosis score; FIB4, fibrosis-4 index; ba-PWV, brachial to ankle pulse wave velocity; ABI, ankle-brachial index; CIMT, carotid intima-media thickness.

We then compared the changes of diabetic complications-related metabolic biomarkers between clusters (**Figure 3** and **Supplementary Table 3**). ACR and eGFR levels worsened in most of the clusters after 4.4 years. Patients assigned to SIDRD presented with the highest ACR level (median 11.0 mg/g at baseline and 14.9 mg/g at follow-up) while participants in MARD showed the lowest eGFR level both at baseline (80.1 ± 12.3 ml/min/1.73m^2^) and follow-up (75.8 ± 13.2 ml/min/1.73m^2^) compared with the other clusters (**Figures 3A, B**). Markers of liver fibrosis are shown in **Figures 3C–E**. The APRI, NFS, FIB4 scores mostly showed significantly increased trend in 4 clusters. After 4.4 years, the ba-PWV, ABI and CIMT increased significantly in SIDRD. The ba-PWV level also increased in MARD and MOD. The CIMT level increased in all the clusters (**Figures 3F–H**).

## Discussion

Using data from a prospective cohort study of Chinese community residents aged ≥ 40 years, we were able to identify four subgroups of T2DM based on the 7 variables measured at baseline and used in the cluster analysis, including age at diagnosis, BMI, FPG, 2hPG, HbA1c, HOMA-β, and HOMA-IR. Clusters were separated well in phenotypic characteristics and presented with different risks of complications. Our study had three key findings. Firstly, we found 2 clusters (SIDRD and SOIRD) which showed different features in Chinese T2DM from those in Ahlqvist-derived diabetes classifications in Caucasians (6, 9). Secondly, after adjustment for multiple covariates, patients in different clusters presented significantly different risks of comprehensive diabetes-related complications. Thirdly, we observed different changes in subclinical metabolic biomarkers and outcome indexes among clusters as the disease progressed after a median of 4.4 years.

Compared with the current classification of diabetes, the novel classification proposed by Ahlqvist et al. in European populations had been verified in different populations and showed advantages in predicting diabetes progression and estimating risks of complications (7–9, 14, 31). The 2hPG level is an important marker for complications in T2DM. Chinese diet is characterized by a higher consumption of carbohydrate-rich cereals, accounting for 60% of the glycemic load (32). Furthermore, our previous study found that the 2hPG level is predictive of diabetes and complications in Chinese adults, independent of FPG and HbA1c (13). However, 2hPG was not included in cluster analysis in previous studies (6, 7, 14). When 2hPG was taken into account in the current study, the SIDRD cluster had a substantially higher 2hPG at diagnosis and at follow-up, and had higher risks of developing CKD than the other clusters. It would be helpful to target these individuals with intensified treatment to reduce 2hPG level and prevent diabetic complications.

Characteristics of the clusters identified in our study showed some differences from those in the Swedish cohort (6), especially the SIDRD and SOIRD clusters. The SIDRD cluster presented with combined insulin deficiency and insulin resistance. The SOIRD cluster was featured as insulin resistant and obese which was different from insulin-resistant SIRD cluster as reported by previous studies (6, 7, 9). The presentation of both insulin-resistant and insulin-deficient features in SIDRD cluster in East Asians, including Chinese, may be due to the ethnic characteristics of Asians with a lower capacity of insulin secretion and a rapidly increased insulin resistance compared to western populations (33, 34).

CVD is the major cause of morbidity and mortality in T2DM, and even in prediabetes (35, 36). Pathophysiological changes involved in the development of CVD in T2DM patients included myocardial insulin resistance, mitophagy, oxidative stress, apoptosis, and inflammation (37–39). The associations between different diabetes clusters and risks of macrovascular and microvascular complications had been reported (6, 9). However, little is known about the relationship between novel diabetes subgroups and the progression of subclinical atherosclerosis. Ba-PWV is an indicator of arterial stiffness and is an independent predictor of CVD risks (40). In the present study, we found that the cluster of MARD was associated with a higher risk of developing abnormal ba-PWV. Moreover, the higher risk of abnormal ABI in the SOIRD cluster indicated that age and insulin resistance might play an important role in the development of CVD (41).

We observed that patients in the SIDRD cluster had the highest risk of developing CKD, while in previous studies the highest risk was in the SIRD cluster accompanied with serious insulin resistance (6, 9). Serious insulin resistance and insulin deficiency in SIDRD contribute to the development of CKD. Insulin resistance was associated with diabetic kidney disease regardless of the HbA1c level (6), while the insulin deficiency speeds up the development of CKD.

Non-alcoholic fatty liver disease (NAFLD) is associated with an increased risk of diabetes, CKD, and cardiovascular disease (42). In 2019, a consensus of international experts recommended to use metabolic (dysfunction) associated MAFLD to raise the awareness of the fatty liver disease (26). Our study benefited from the use of B-ultrasound to diagnose MAFLD in diabetes patients. We found obesity-related clusters, including MOD and SOIRD, had higher risks of developing MAFLD compared with the other clusters. Whereas, in the study of Ahlqvist et al., the NAFLD was associated with SIRD but not with MOD (6). This might due to the cross-ethnic differences and that Chinese people generally had higher amounts of visceral fat than White people (43). Furthermore, non-invasive liver fibrosis indexes have been used to stratify the risk of liver-related morbidity and mortality in MAFLD patients with comparable performance to a liver biopsy. The MARD cluster had the highest risk of liver fibrosis observed by FIB-4 in our study, indicating that patients with MAFLD in the MARD cluster should pay more attention to monitoring liver fibrosis.

Our study has several limitations. Firstly, parameters such as the glutamic acid decarboxylase antibodies (GADA) were not measured or included in the cluster analysis in the current study. However, the prevalence of being GADA-positive could be less than 5.9% in population-based screening of adult-onset T2DM in China (7). Secondly, the follow-up duration was short and the limited numbers of incident macrovascular and microvascular diseases might not be able to provide sufficient statistical power to detect a true difference, therefore a further examination of these diabetic complications were not conducted. Thirdly, our study was conducted in community residents in Shanghai, China, which had limited extensibility to other populations.

In conclusion, the middle-aged and elderly Chinese adults with newly-diagnosed diabetes can be allocated to specific clusters. Two subgroups SIDRD and SOIRD in the current study showed higher risks of developing diabetes-related complications. In addition, obesity-related groups of MOD and SOIRD presented with a higher incidence of MAFLD. The more precise classifications of Chinese diabetes patients can aid in precision treatment of diabetes and prevention of complications.

## Data Availability Statement

The IRB has requested that currently, the dataset should be used by the research team members only. If the dataset has to be accessed to verify the results, the request can be directed to the corresponding author. Requests to access the datasets should be directed to Yu Xu, jane.yuxu@gmail.com.

## Ethics Statement

The studies involving human participants were reviewed and approved by The Institutional Review Board of Ruijin Hospital, Shanghai Jiaotong University School of Medicine. The patients/participants provided their written informed consent to participate in this study.

## Author Contributions

FW, RZ, LL, YX, GN, and WC conceived and designed the study. FW, RZ, and LL analyzed and interpreted the data. FW and RZ drafted the manuscript. YX, GN, and WC revised it. MX, JL, ZZ, ML, TW, SW, YB, and YX collected data. FW, RZ, and YX are the guarantors of this work and, as such, had full access to all the data in the study and take responsibility for the integrity of the data and the accuracy of the data analysis. All authors agreed to be accountable for all aspects of the work and approved the final version of the paper.

## Funding

This work was supported by the grants from the National Natural Science Foundation of China (81870560, 81700764, 81941017, 81770842), the Shanghai Municipal Government (18411951800), Chinese Academy of Medical Sciences (2018PT32017, 2019PT330006), the Shanghai Shenkang Hospital Development Center (SHDC12019101, SHDC2020CR1001A, SHDC2020CR3069B), the Shanghai Jiaotong University School of Medicine (DLY201801), and the Ruijin Hospital (2018CR002).

## Conflict of Interest

The authors declare that the research was conducted in the absence of any commercial or financial relationships that could be construed as a potential conflict of interest.

## Publisher’s Note

All claims expressed in this article are solely those of the authors and do not necessarily represent those of their affiliated organizations, or those of the publisher, the editors and the reviewers. Any product that may be evaluated in this article, or claim that may be made by its manufacturer, is not guaranteed or endorsed by the publisher.
