# Supplementary material for: Novel Subgroups and Chronic Complications of Diabetes in Middle-Aged and Elderly Chinese:A Prospective Cohort Study
Source: Front Endocrinol (Lausanne). 2022 Jan 26;12:802114. doi: 10.3389/fendo.2021.802114 (PMC8825378; doi:10.3389/fendo.2021.802114)
Supplement: Supplementary file 1 [file DataSheet_1.docx]

**SUPPLEMENTAL MATERIALS**

Supplementary Table 1. Cluster center coordinates for the cluster analysis using baseline variables

Supplementary Table 2. Changes of participants’ characteristics from baseline to follow-up

Supplementary Table 3. Progression of diabetes-related complications index from baseline to follow-up

Supplementary Figure 1. Flowchart of study population

Supplementary Table 1. Cluster center coordinates for the cluster analysis using baseline variables

| Cluster | Age-onset | BMI | HbA1c | FPG | 2hPG | HOMA-IR | HOMA-β |
| --- | --- | --- | --- | --- | --- | --- | --- |
| MARD | 0.1570654 | 0.945656875 | -0.28804906 | -0.08114256 | -0.2422876 | 1.1037617 | 1.4310780 |
| MOD | -0.6893855 | 0.116841386 | -0.05442841 | -0.17359253 | -0.1040522 | -0.2043360 | -0.2554849 |
| SOIRD | -0.1582059 | -0.003504711 | 2.74622102 | 2.45620798 | 2.7547589 | 1.0188340 | -0.8500214 |
| SIDRD | 0.7762945 | -0.650341413 | -0.40620411 | -0.30922974 | -0.3735039 | -0.5848721 | -0.2738821 |

Abbreviations: BMI, body mass index; HbA1c; hemoglobin A1c; FPG, fasting plasma glucose; 2hPG, 2 hours post-load plasma glucose; HOMA-IR, homoeostatic model assessment estimates of insulin resistance; HOMA-β, homoeostatic model assessment estimates of β-cell function; MARD, mild age-related diabetes; MOD, mild obesity-related diabetes; SIDRD, severe insulin-deficient and insulin-resistant diabetes; SOIRD, severe obesity-related and insulin-resistant diabetes.

Supplementary Table 2. Changes of participants’ characteristics from baseline to follow-up

|  | MARD (n=213) | | |  | MOD (n=294) | | |  | SIDRD (n=61) | | |  | SOIRD (n=120) | | |
| --- | --- | --- | --- | --- | --- | --- | --- | --- | --- | --- | --- | --- | --- | --- | --- |
|  | Baseline | Follow-up | *P* |  | Baseline | Follow-up | *P* |  | Baseline | Follow-up | *P* |  | Baseline | Follow-up | *P* |
| SBP (mmHg) | 149.3 (18.1) | 141.0 (16.2) | <0.001 |  | 146.8 (19.7) | 139.1 (17.0) | <0.001 |  | 149.3 (19.1) | 143.0 (17.8) | 0.047 |  | 151.5 (20.9) | 142.3 (18.1) | <0.001 |
| DBP (mmHg) | 81.6 (10.0) | 74.6 (9.3) | <0.001 |  | 87.6 (10.5) | 79.0 (10.2) | <0.001 |  | 86.9 (10.6) | 77.6 (8.7) | <0.001 |  | 84.9 (11.0) | 75.8 (10.4) | <0.001 |
| FPG (mmol/L) | 6.1 (1.0) | 6.9 (1.4) | <0.001 |  | 6.9 (1.2) | 7.5 (1.8) | <0.001 |  | 12.9 (2.8) | 9.0 (3.2) | <0.001 |  | 6.4 (1.2) | 7.16 (1.7) | <0.001 |
| 2hPG (mmol/L) | 12.1 (3.0) | 12.6 (3.8) | 0.0884 |  | 12.8(3.2) | 13.1 (4.2) | 0.3512 |  | 25.5 (4.4) | 15.3 (5.0) | <0.001 |  | 13.7 (3.1) | 13.24 (3.8) | 0.2463 |
| HbA1c (%) | 6.2 (0.7) | 6.1 (0.8) | 0.1876 |  | 6.5 (0.8) | 6.4 (1.0) | 0.0063 |  | 10.6 (1.6) | 7.4 (1.7) | <0.001 |  | 6.4 (0.8) | 6.40 (1.1) | 0.8416 |
| LDL (mmol/L) | 3.29 (0.88) | 3.56 (0.81) | <0.001 |  | 3.33 (0.91) | 3.65 (0.82) | <0.001 |  | 3.39 (1.10) | 3.50 (0.78) | 0.2894 |  | 3.44 (0.87) | 3.60 (0.75) | 0.0269 |
| HDL (mmol/L) | 1.33 (0.31) | 1.34 (0.32) | 0.4011 |  | 1.24 (0.29) | 1.26 (0.25) | 0.0316 |  | 1.20 (0.30) | 1.17 (0.25) | 0.2989 |  | 1.24 (0.27) | 1.27 (0.27) | 0.0904 |
| TG (mmol/L) | 1.56 (1.20, 2.06) | 1.49 (1.11, 1.98) | 0.6856 |  | 1.71 (1.22, 2.54) | 1.75 (1.22, 2.56) | 0.8703 |  | 2.25 (1.49, 3.40) | 1.70 (1.20, 2.55) | 0.0068 |  | 1.92 (1.45, 2.53) | 1.75 (1.36, 2.44) | 0.0255 |
| TC (mmol/L) | 5.49 (1.02) | 5.27 (1.07) | <0.001 |  | 5.55 (1.05) | 5.32 (1.05) | <0.001 |  | 5.91 (1.29) | 5.04 (0.95) | <0.001 |  | 5.62 (0.96) | 5.23 (0.92) | <0.001 |
| ApoB / ApoA1 | 0.80 (0.22) | 0.70 (0.17) | <0.001 |  | 0.90 (0.26) | 0.72 (0.17) | <0.001 |  | 0.95 (0.29) | 0.74 (0.18) | <0.001 |  | 0.89 (0.24) | 0.72 (0.16) | <0.001 |

Data are mean (SD) for normal variables or median ( IQR) for skewed variables and n (%) for categorical variables. Paired T-test was used to evaluate the difference between baseline and follow-up levels.

Abbreviations: SBP, systolic blood pressure; DBP, diastolic blood pressure; FPG, fasting plasma glucose; 2hPG, 2h post-load plasma glucose; HbA1c, hemoglobin A1c; LDL, low-density lipoprotein; HDL, high-density lipoprotein; TG, triglycerides; TC, total cholesterol; ApoB, apolipoprotein B; ApoA1, apolipoprotein A1; MARD, mild age-related diabetes; MOD, mild obesity-related diabetes; SIDRD, severe insulin-deficient and insulin-resistant diabetes; SOIRD, severe obesity-related and insulin-resistant diabetes.

Supplementary Table 3. Progression of diabetes-related complications index from baseline to follow-up

|  | MARD (n=213) | | |  | MOD (n=294) | | |  | SIDRD (n=61) | | |  | SOIRD (n=120) | | |
| --- | --- | --- | --- | --- | --- | --- | --- | --- | --- | --- | --- | --- | --- | --- | --- |
|  | Baseline | Follow-up | *P* |  | Baseline | Follow-up | *P* |  | Baseline | Follow-up | *P* |  | Baseline | Follow-up | *P* |
| MAFLD, n (%) | 67 (31.5) | 59 (27.7) | 0.3186 |  | 166 (56.5) | 152 (51.7) | 0.1962 |  | 40 (65.6) | 27 (44.3) | 0.0021 |  | 99 (82.5) | 83 (69.2) | 0.0179 |
| APRI | 0.27 (0.20, 0.33) | 0.34 (0.27, 0.48) | <0.001 |  | 0.24 (0.20, 0.34) | 0.33 (0.26, 0.46) | <0.001 |  | 0.28 (0.19, 0.46) | 0.33 (0.26, 0.48) | 0.3335 |  | 0.32 (0.24, 0.61) | 0.42 (0.32, 0.67) | <0.001 |
| NFS | -0.52 (1.00) | 0.24 (0.95) | <0.001 |  | -1.17 (0.91) | -0.45 (0.87) | <0.001 |  | -0.92 (1.22) | -0.09 (1.16) | <0.001 |  | -0.56 (1.19) | 0.25 (1.43) | <0.001 |
| FIB4 | 1.97 (1.61) | 2.49 (1.06) | <0.001 |  | 1.30 (0.61) | 1.80 (0.83) | <0.001 |  | 1.63 (1.09) | 2.56 (4.03) | 0.077 |  | 1.82 (1.25) | 2.80 (2.57) | <0.001 |
| eGFR (ml/min/1.73m^2^) | 80.1 (12.3) | 75.8 (13.2) | <0.001 |  | 91.5 (11.5) | 86.8 (11.9) | <0.001 |  | 84.1 (13.3) | 81.3 (13.7) | 0.0472 |  | 83.7 (12.0) | 80.5 (12.9) | <0.001 |
| ACR (mg/g) | 5.6 (3.3, 10.6) | 11.7 (7.8, 20.5) | 0.0994 |  | 5.4 (2.9, 13.1) | 9.8 (6.1, 18.1) | 0.0601 |  | 11.0 (4.1, 20.1) | 14.9 (7.7, 30.2) | 0.025 |  | 6.9 (3.8, 14.5) | 13.1 (7.8, 23.4) | 0.016 |
| ABI | 1.11 (0.11) | 1.10 (0.22) | 0.0884 |  | 1.09 (0.10) | 1.10 (0.22) | <0.001 |  | 1.08 (0.08) | 1.14 (0.08) | <0.001 |  | 1.08 (0.11) | 1.06 (0.30) | <0.001 |
| ba-PWV (cm/s) | 1863.7 (470.1) | 1948.20 (391.6) | <0.001 |  | 1643.95 (326.3) | 1689.20 (320.7) | 0.0148 |  | 1780.90 (414.4) | 1919.93 (376.5) | 0.012 |  | 1795.70 (372.2) | 1823.80 (381.0) | 0.3482 |
| CIMT (mm) | 0.62 (0.12) | 0.75 (0.16) | <0.001 |  | 0.58 (0.10) | 0.71 (0.15) | <0.001 |  | 0.60 (0.12) | 0.74 (0.18) | <0.001 |  | 0.60 (0.14) | 0.73 (0.14) | <0.001 |

Data are mean (SD) for normal variables or median ( IQR) for skewed variables and n (%) for categorical variables. Paired T-test was used to evaluate the difference between baseline and follow-up levels.

Abbreviations: MAFLD, metabolic associated fatty liver disease; APRI, aspartic acid aminotransferase (AST) and platelet (PLT) ratio; NFS, nonalcoholic fatty liver disease fibrosis scores; FIB-4, fibrosis 4 score; eGFR, estimated glomerular filtration rate; ACR, albumin-to-creatinine ratio; ABI, ankle brachial index; ba-PWV, brachial-ankle pulse wave conduction velocity; CIMT, carotid intima-media thickness; MARD, mild age-related diabetes; MOD, mild obesity-related diabetes; SIDRD, severe insulin-deficient and insulin-resistant diabetes; SOIRD, severe obesity-related and insulin-resistant diabetes.

Supplementary Figure 1. Flowchart of study population

Jiading baseline population in 2010 (n=10375)

6 were excluded by missing measures of baseline cluster analysis variables

703 were followed up

427 did not participate in the follow-up visit

1130 eligible individuals included at baseline for cluster analysis

1136 were newly diagnosed diabetes by ADA 2010 criteria

15 were excluded by missing measures of follow-up cluster analysis variables

688 were included in analysis

23 with CVD history at baseline were excluded and:

177 were further excluded from analysis of **ba-PWV**

150 had abnormal ba-PWV at baseline

27 did not have ba-PWV data at baseline or at follow-up

136 were further excluded from analysis of **ABI**

120 had abnormal ABI at baseline

16 did not have ABI data at baseline or at follow-up

160 were further excluded from analysis of **CIMT**

160 had abnormal CIMT at baseline

181 were further excluded from analysis of incident **CKD**

36 had CKD history at baseline

72 had abnormal eGFR or ACR at baseline

73 did not have eGFR or ACR data at baseline or at follow-up

408 were further excluded from analysis of incident **MAFLD**

372 had MAFLD at baseline

36 did not have MAFLD data at follow-up

408 were further excluded from analysis of increased **FIB4**

359 had increased FIB4 at baseline

49 did not ha**ve** FIB4 related measures at follow-up

127 were further excluded from analysis of increased **APRI**

109 had increased APRI at baseline

18 did not have APRI related measures at follow-up

521 were further excluded from analysis of increased **NFS**

485 had increased NFS at baseline

36 did not have NFS related measures at follow-up

MAFLD and liver fibrosis

280 were analyzed for incident MAFLD

280 were analyzed for FIB4

561 were analyzed for APRI

167 were analyzed for NFS

507 were analyzed for incident CKD

Atherosclerosis

488 were analyzed for ba-PWV

529 were analyzed for ABI

505 were analyzed for CIMT
